# A New Microfluidic Device for Classification of Microalgae Cells Based on Simultaneous Analysis of Chlorophyll Fluorescence, Side Light Scattering, Resistance Pulse Sensing

**DOI:** 10.3390/mi7110198

**Published:** 2016-11-02

**Authors:** Junsheng Wang, Jinsong Zhao, Yanjuan Wang, Wei Wang, Yushu Gao, Runze Xu, Wenshuang Zhao

**Affiliations:** 1 College of Information and Science Technology, Dalian Maritime University, Dalian 116026, China; zhaojinsong9@gmail.com (J.Z.); wswl408@gmail.com (Y.W.); zwldmm@gmail.com (W.W.); ICMFLOC@dlmu.edu.cn (Y.G.); dlmuxwg@gmail.com (R.X.); zzlswl1@gmail.com (W.Z.); 2 Collaborative Innovation Center for Vessel Pollution Monitoring and Control, Dalian Maritime University, Dalian 116026, China

**Keywords:** microalgae cells classification, ballast water, microfluidic chip

## Abstract

Fast on-site monitoring of foreign microalgae species carried by ship ballast water has drawn more and more attention. In this paper, we presented a new method and a compact device of classification of microalgae cells by simultaneous detection of three kinds of signals of single microalgae cells in a disposable microfluidic chip. The microfluidic classification device has advantages of fast detection, low cost, and portability. The species of a single microalgae cell can be identified by simultaneous detection of three signals of chlorophyll fluorescence (CF), side light scattering (SLS), and resistance pulse sensing (RPS) of the microalgae cell. These three signals represent the different characteristics of a microalgae cell. A compact device was designed to detect these three signals of a microalgae cell simultaneously. In order to demonstrate the performance of the developed system, the comparison experiments of the mixed samples of three different species of microalgae cells between the developed system and a commercial flow cytometer were conducted. The results show that three kinds of microalgae cells can be distinguished clearly by our developed system and the commercial flow cytometer and both results have good agreement.

## 1. Introduction

Biological invasions brought by ship ballast water have caused serious effects to local ecological environments and human health [1,2,3,4]. Microalgae are the most common species exiting in ballast water and the main object for ship ballast water treatment and detection. Microalgae species identification is very important for ballast water treatment. Different species of microalgae cells may need different treatment methods or different doses of the same treatment method [5,6]. The survived microalgae in the treated ballast water also need to be further classified. Some harmful microalgae species need stricter treatments, while harmless ones do not need to be killed. Furthermore, according to the International Convention for the Control and Management of Ships’ Ballast Water and Sediments, the detection of microalgae species for ballast water after being treated should not delay the normal navigation of ships. Therefore, fast on-site identification of microalgae species is necessary for ballast water detection. 

The most traditional method for monitoring microalgae species is by using a microscope. Microalgae species are judged by the human eye and this method needs well-trained personnel, and is laborious and inaccurate (i.e., the identification results often depend on the operator’s experience) [7,8]. Automatic monitoring methods mainly include optical imaging, absorption spectrum, and flow cytometry [9,10,11,12,13,14,15]. In the optical imaging method, microalgae cells are classified through image matching analysis and it is mainly applied for stationary objects, otherwise it will require high speed cameras. High-performance computer processing and real-time image processing algorithms are also key factors limiting the application of this method. In the absorption spectrum method, spectrophotometers are usually used to determine pigment compositions in microalgae cells, but they cannot count cells. Moreover, the commercial spectrophotometers are usually bulky and expensive. Flow cytometers can accurately identify microalgae species and count cells, but commercial flow cytometers involve complex operational procedures and are expensive and bulky. These factors limit their application in field and on-site detection. 

The lab-on-a-chip (LOC) technique has many unique advantages for fast on-site detection, such as a small sample size, fast detection, low cost, and portability. It has been applied widely in biology, environmental science, chemistry, food, and medicine [16,17,18,19,20,21,22,23,24,25]. Some LOC-based microalgae identification methods and devices have been presented in the past few years [26,27,28,29,30]. The typical characteristics of these devices include using a microfluidic chip integrated with one or more optical fibers. There are two main reasons of integrating an optical fiber in a microfluidic chip. One reason is to improve excitation efficiency and emission light collection efficiency because the optical fiber can be put very close to the light source and cells. The other reason is to converge light to a small spot. For example, the method of forward scattering light (FSC), which is usually used to determine the cell’s size, needs the light spot size close to the size of the cells by using optical fibers. However, this method of using optical fibers has also some disadvantages, such as high cost, complex fabrication, and poor replicability and stability. Compared with this method of optical fibers, the discrete optical layout can simplify the fabrication process of the microfluidic chips, reduce costs, improve replicability, and is more suitable for rapid on-site detection in ships.

Therefore, in this paper, we developed a new microalgae identification method and a compact system of using a disposable microfluidic chip and simultaneous detection of three kinds of signals of single microalgae cells: chlorophyll fluorescence (CF), side light scattering (SLS), and resistance pulse sensing (RPS). The microalgae cells flow one by one along the microchannel in a disposable microfluidic chip. When a single microalgae cell is passing through the detection spot, the CF, SLS, and RPS signals of the microalgae cell are detected simultaneously. Through the analysis of these three signals, the microalgae cell can be distinguished into the different species. The intensities of CF and SLS of single microalgae cells are detected with high sensitivity by the shift phase differential amplifier developed in this work. To demonstrate the performance of the presented method and the detection system, the comparison experiments of mixed samples of three different species of microalgae cells were conducted by using the developed system and a commercial flow cytometer.

## 2. Materials and Methods

### 2.1. Classification Principle of Microalgae Cells

**Figure 1:**
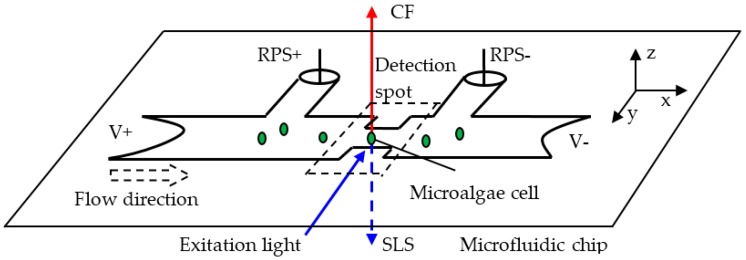
Illustration of the principle of classification of single microalgae cells based on the simultaneous detection of CF, SLS and RPS signals.

The principle of classification of single microalgae cells in this paper is shown in Figure 1. When a single microalgae cell is passing through the detection spot, the microalgae cell is excited by the incident excitation light with ~480 nm wavelength along the negative *y* axis, three kinds of signals of CF, SLS, and RPS of the microalgae cell are detected simultaneously. The resulting CF of the microalgae cell with ~680 nm wavelength is detected from the positive *z* axis and SLS with ~480 nm wavelength is detected from the negative *z* axis. Meanwhile, the signal of RPS of the microalgae cell based on the Coulter principle is acquired from the difference of two sense arms of RPS+ and RPS−. These three signals of single microalgae cells correspond to three different characteristics of the microalgae cell, respectively. CF is used to represent the activity of a microalgae cell; SLS can be used to characterize the intracellular contents and the size and surface roughness of the cell; and the size of a microalgae cell can be determined by RPS. These three characteristics of single microalgae cells are required to be detected in the international convention of ballast water treatment.

#### 2.1.1. A Living Microalgae Cell Determination by CF Intensity

It is particularly important to judge if a microparticle is a living microalgae cell or not, for the microalgae cells species identification in ship’s ballast water. Chlorophyll fluorescence (CF) comes from excess energy emission in the photosynthesis of plants and there exists a strong relationship between chlorophyll fluorescence and the photosynthesis electron transport chain. Chlorophyll fluorescence intensity has been proved to be positively correlated with chlorophyll contents in cells. More importantly, chlorophyll fluorescence can be used to evaluate the photosynthesis capacity and activity of plant cells [31,32,33]. Theoretically, the photosynthetic activity of microalgae cells can be represented by the net photosynthetic rate, which is proportional to the photosynthesis electron transport rate in Photosynthesis System II. This can be expressed by Equation (1):

(1) $$P_{n}\propto k_{I}\times r_{e}$$

Here, *P_n_* is the net photosynthetic rate, *r_e_* is the photosynthesis electron transport rate in Photosynthesis System II, *k_I_* is a coefficient related to the intercellular concentration of CO_2_ and the CO_2_ compensation point in the absence of mitochondria respiration. On the other hand, the intensity of chlorophyll fluorescence is proportional to the photosynthesis electron transport rate in Photosynthesis System II. Thus, we can obtain the following Equation (2):

(2) $$I_{CF}\propto k_{r}\times r_{e}\propto \;\frac{k_{r}}{k_{I}}\times P_{n}$$

Here, *I_CF_* is the intensity of chlorophyll fluorescence, *k_r_* is a fixed scale factor related to energy conversion efficiency in the photosynthesis electron transport chain. *k_I_* is usually a constant under the conditions of a given excitation light, temperature, and CO_2_ concentration. Therefore, in this paper, the intensity of chlorophyll fluorescence is used to represent the activity of single microalgae cells and then determine whether a microparticle in ballast water is a living microalgae cell or not. 

#### 2.1.2. Contents Characteristics in a Microalgae Cell by SLS Intensity

When light is irradiated onto an object, light side scattering occurs, which is related to the intracellular contents, size, and surface roughness, and has a wide range of applications in biological, chemical, physical, optical, and other fields [34,35,36,37,38]. According to Mie scattering theory, when a cell is irradiated by a beam of light with light intensity *I*_0_ and wavelength λ, the light intensity *I*_θ_ in the direction of scattering angle θ can be expressed by the following Equation (3):

(3) $$I\left(\theta \right)=I_{0}\frac{\lambda^{2}}{8\pi^{2}r^{2}}\left(i_{1}+i_{2}\right)$$

Here, *r* is the distance between the particle or cell and detection spot, (*i*_1_ + *i*_2_) is an intensity function shown, as in Equation (4):

(4) $$i_{1}+i_{2}=f\left(\frac{\pi d}{\lambda },m,\theta \right)$$

This intensity function (*i*_1_ + *i*_2_) is related to the parameters including excitation wavelengh λ, scattering angel θ, the particle diameter *d*, and relative refractive index *m*. In this study, λ and θ are fixed and while the relative refractive index m depends on the intracellular contents and surface roughness, which cannot be expressed accurately by a mathematical function, the light intensity *I*_θ_ can only be determined by experiments.

#### 2.1.3. Size Representation of a Microalgae Cell by RPS

As shown in Figure 1, when a microalgae cell enters the detection spot, the electrical resistance *R* of the area around the detection spot will change and according to Coulter principle [39,40], the change of electrical resistance Δ*R* is related to the parameters including the resistance of the buffer solution, the size of the microalgae cell *d*, the width *D*, and length *L* of the detection spot. When the structure of the microfluidic chip and the buffer solution are fixed, the value of ΔR is proportional to the size of the microalgae cell:

(5) ΔR ∝ d

The change of Δ*R* will lead to the change of the voltage of the upstream *V*_RPS+_ and downstream *V*_RPS−_ of the detection spot, which can be expressed by the following Equations (6) and (7):

(6) $$\Delta V_{\mathrm{RPS}+}=+\frac{R_{1}\Delta R}{R_{s}^{2}+R_{s}\Delta R}\left(V_{-}-V_{+}\right)$$

(7) $$\Delta V_{\mathrm{RPS}-}=-\frac{R_{3}\Delta R}{R_{s}^{2}+R_{s}\Delta R}\left(V_{-}-V_{+}\right)$$

Here, $R_{s}=R_{1}+R_{2}+R_{3}$, where *R*_1_, *R*_2_, and *R*_3_ are the upstream, detection spot, and downstream resistances in the microchannel, respectively. 

Thus, we can obtain Equation (8) from Equations (6) and (7):

(8) $$\Delta V_{\mathrm{RPS}}=\Delta V_{\mathrm{RPS}+}-\Delta V_{\mathrm{RPS}+}=\frac{\left(R_{1}+R_{3}\right)\Delta R}{R_{s}^{2}+R_{s}\Delta R}\left(V_{-}-V_{+}\right)$$

From Equation (8), it can be seen that Δ*V*_RPS_ is proportional to Δ*R* which, in turn, is proportional to the size of a microalgae cell d. Thus, the size of the microalgae cell can be determined by measuring the voltage difference between *V*_RPS+_ and *V*_RPS−_.

If two microalgae cells belong to the same category of microalgae, they will have similar chlorophyll and cell contents, as well as similar surface roughness and sizes. In this study, the species of microalgae cells will be identified by these characteristics detected by the self-developed compact system in our lab.

### 2.2. Simultaneous Detection System for Signals of CF, SLS, and RPS

**Figure 2:**
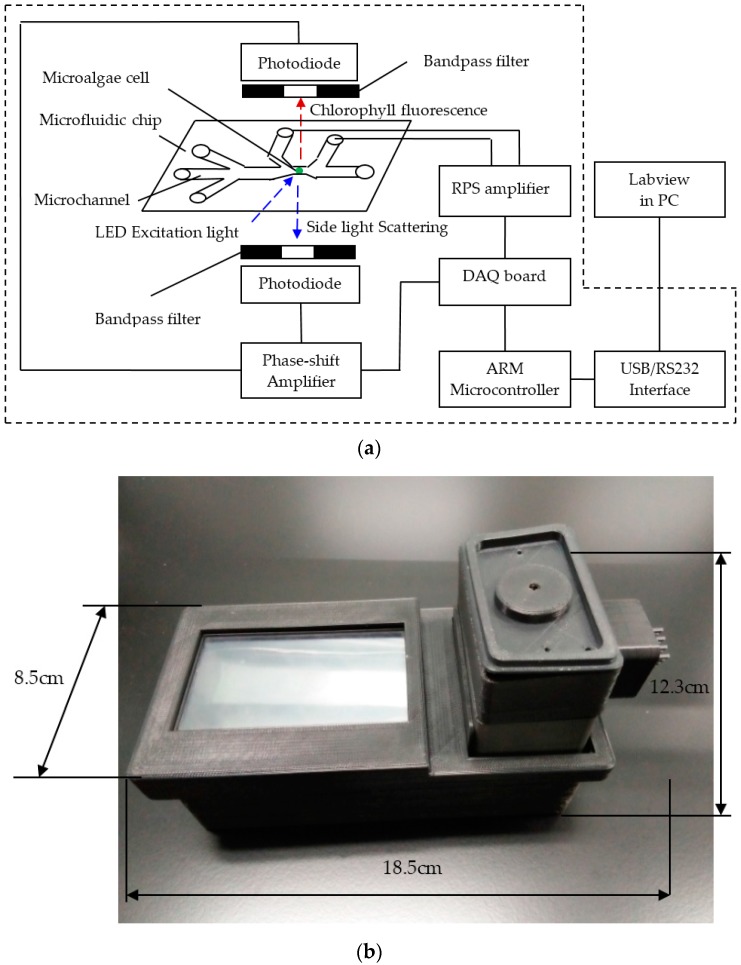
The schematic diagram and photos of the detection system of CF, SLS, and RPS signals of single microalgae cells: (**a**) the schematic diagram of the detection system; and (**b**) a photo of the detection system.

As mentioned before, a microalgae cell can be classified by simultaneous detection of three different signals of CF, SLS, and RPS of the microalgae cell. The schematic diagram of the detection system is shown in Figure 2a. The detection system is mainly comprised of a microfluidic chip, an excitation light source, photodetectors, signal amplifiers, data acquisition and processing, a microcontroller, and a data interface. All of these parts are integrated in a compact mechanical structure. The photos of the detection system are shown in Figure 2b. A microfluidic chip provides a sample platform for microalgae cells and the microalgae cells will flow along the designed microchannels in the microfluidic chip. An LED (LZ1-10B200, LED Engin, Inc., San Jose, CA, USA) was chosen as the excitation light source and the CF and SLS of the microalgae cell are generated simultaneously under the excitation of LED light when a microalgae cell is passing through the detection spot. The CF and SLS of the microalgae cell are detected by two photodiodes (S8745, Hamamatsu, Bridgewater, NJ, USA) along the positive *z* axis and the negative *z* axis, respectively. According to the excitation and emission spectra of CF of microalgae cells, an emission filter (ET680, Chroma, Bellows Falls, VT, USA) is placed between the microfluidic chip and the photodiode at the positive *z* axis direction. In order to block other stray light, an emission filter (ET470, Chroma, Bellows Falls, VT, USA) is mounted between the microfluidic chip and the photodiode at the negative *z* axis direction. A shift phase differential amplifier was designed to detect the pulse signals of CF and SLS from the photodiodes. Meanwhile, two microchannels are designed as RPS arms at both ends of the detection spot in the microfluidic chip. Through these two sensing arms, the RPS signal of the microalgae cell is processed further by the differential amplifier. An Acorn RISC Machine (ARM) development board with Linux OS (OK6410, Forlinx Embedded Technology, Inc., Baoding, Hebei, China) is used for data acquisition and processing. The RS232 and USB interfaces between ARM board and PC is set up for data communication. Labview software (National Instruments, Austin, TX, USA) on the PC is used for data saving and display. 

### 2.3. Microfluidic Chip Design and Microfabrication

**Figure 3:**
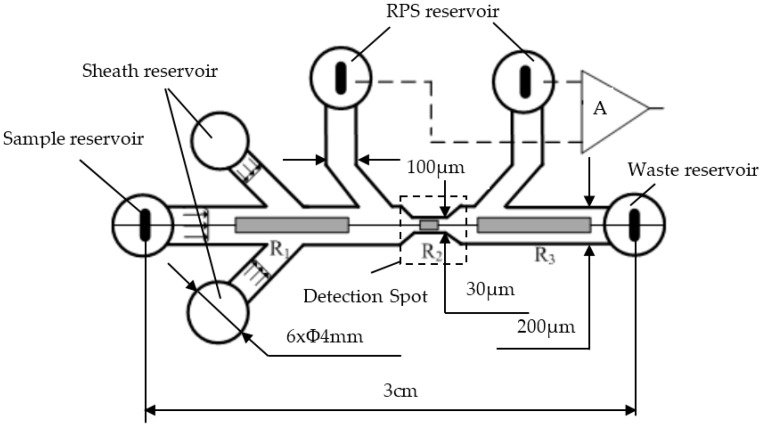
The diagram of the structure and dimensions of the microfluidic chip.

The structural diagram of the designed microfluidic chip is shown in Figure 3. This microfluidic chip has six reservoirs, including one sample reservoir, one waste reservoir, two sheath reservoirs, and two RPS reservoirs. The microalgae cell samples are put into the sample reservoir and phosphate buffer saline (PBS) buffer is placed into two sheath reservoirs for hydrodynamic focusing. The two laminar flow streams from the two sides will force the microalgae cells sample to move in a single line, and pass through the detection spot one by one along the main microchannel center line. The detection spot is at the sense gate where the signals of CF, SLS, and RPS of the microalgae cells are detected simultaneously. The width and length of the main microchannel from the sample reservoir to the waste reservoir junction is 200 µm and 3 cm, respectively. The width of the sheath microchannel and RPS sensing arms are both 100 µm, and the width of the sensing gate is 30 µm. All of the microchannels are 30 µm high and all reservoirs have a diameter of 4 mm and a depth of 2 mm.

The microfluidic chip was fabricated by bonding a polydimethylsiloxane (PDMS) plate with a glass slide (24 mm × 50 mm × 0.15 mm, Citotest Labware Manufacturing Co., Ltd., Haimen, China) by the following standard soft-lithography protocol [41]: A layer of SU-8 photoresist (MicroChem Co., Newton, MA, USA) was spread on a bare silicon wafer (Lijing Co., Ltd., Quzhou, China) by a spin coater (G3P-8, Cookson Electronics Equipment, Indianapolis, IN, USA). Then a photomask containing the designed microchannel pattern was mounted on the silicon wafer and excited with an OAI 150 illuminator. The SU8 master was attained after post-baking and developing processes. Liquid PDMS and curing agent were mixed, degassed, and poured on the master, and then heated at 75 °C for 5 h in a vacuum oven (Isotempmodel 280A, Fisher Scientific, Pittsburgh, PA, USA) under normal pressure. Finally, the PDMS duplicate was taken from the master. Wells were formed by punching holes on the PDMS layer. The PDMS layer with the microchannel pattern was bound onto a glass slide after being treated for 60 s in a plasma cleaner (PDC-30G, Harrick Plasma, Ithaca, NY, USA). 

### 2.4. Sample Preparation

#### 2.4.1. Culture of Microalgae Cells

The microalgae species (*Chlorella vulgaris*, *Isochrysis galbana*, *Dunaliella salina*, *Pyramidomonas delicatula*, and *Tetraselmis chui*) were acquired from Liaoning Sea Fisheries Research Institute in Dalian, China. Each microalgae species was cultured individually in a conical flask of the enriched seawater medium [42], which was shaken one time every three hours. They grew in a CO_2_ thermotank (MGC-300A, Yiheng Technical Co., Ltd., Dalian Maritime University (DMU), Dalian, China) under a photoperiod of 12 h. The temperature was kept at 23 °C and the illumination intensity was 3000–3500 cd/m^2^ in the thermotank. 

#### 2.4.2. Treatments and Standard Assays of Microalgae Cells

The microalgae cells were inactivated and maintained integrity by the water bath method and the operational process follows the standard protocol [43]. The activities of the microalgae cells were determined by using a commercial microscope (Ti-FLC-E, NIKON Corp., Tokyo, Japan). The standard assays of the classification of microalgae species were accomplished by a commercial flow cytometer (FACSCaliburTM Cytometer, BD Biosciences, San Jose, CA, USA). The operation of the commercial microscope and flow cytometer were conducted according to the standard operational instructions. 

## 3. Results and Discussion

### 3.1. The Signals of CF, SLS, and RPS of the Single Microparticles

**Figure 4:**
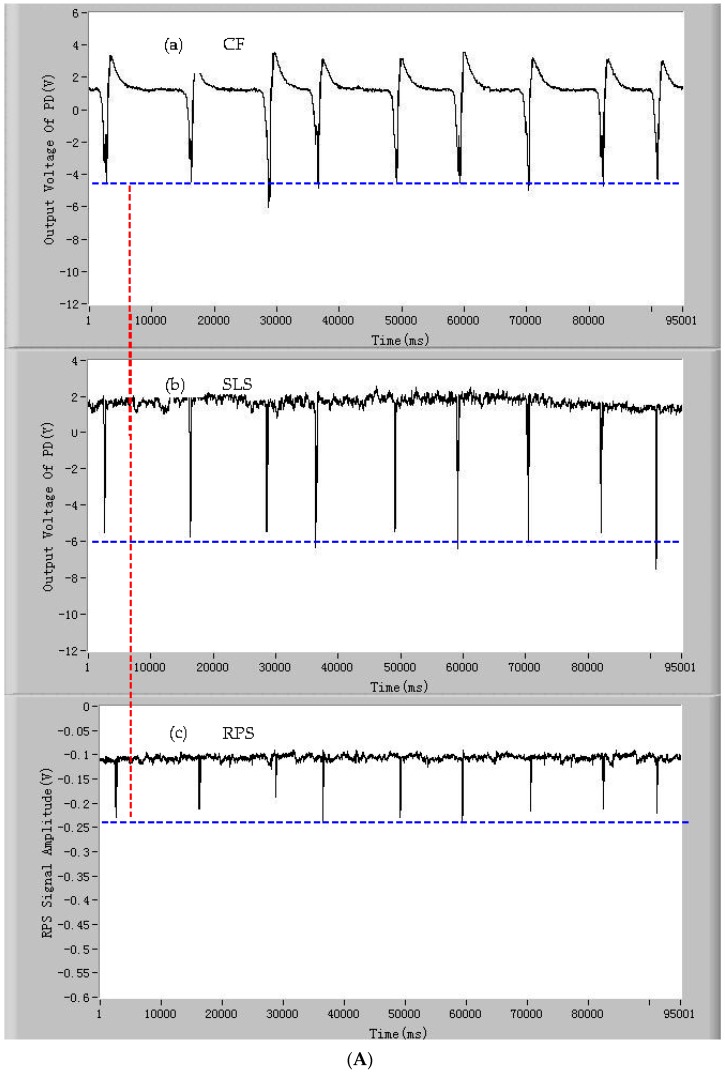

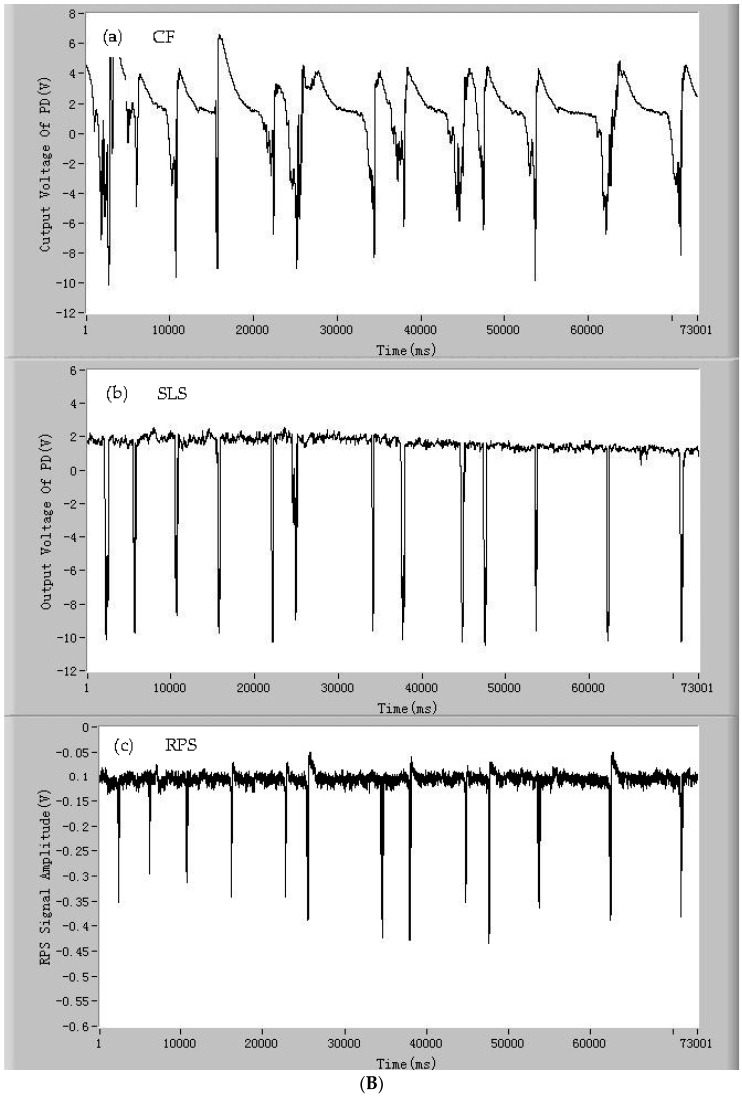

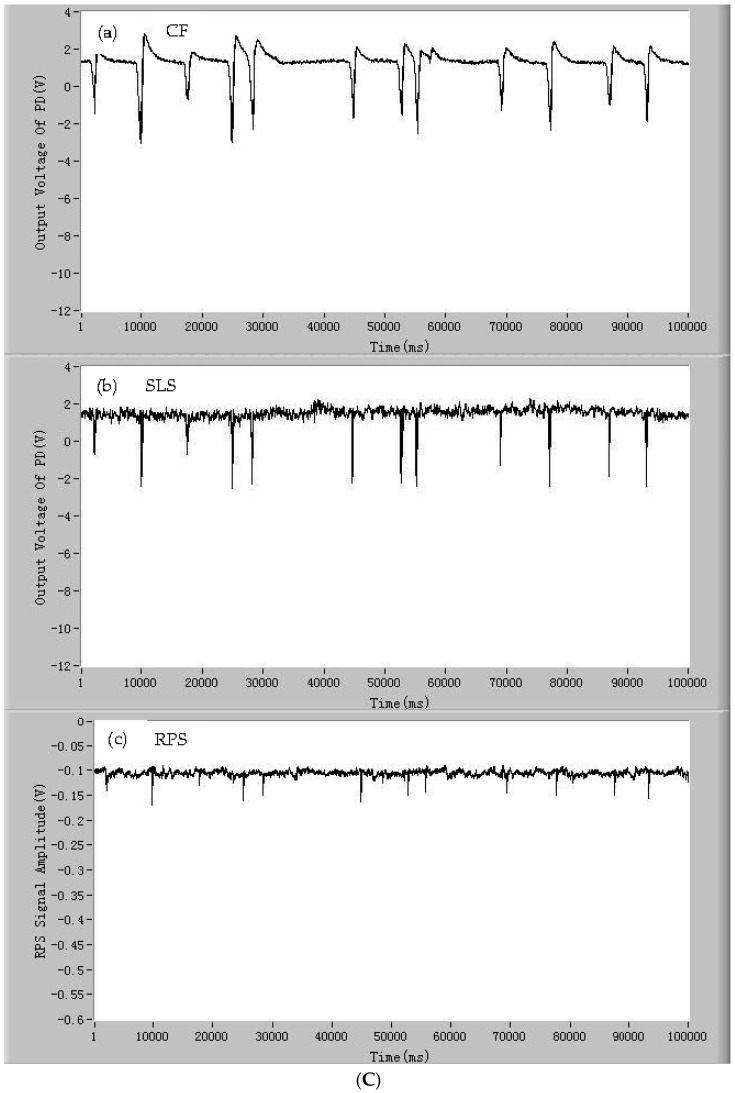
The signals of (**A**) individual living *Dunaliella salina* cells: (**a**) chlorophyll fluorescence (CF); (**b**) side light scattering (SLS); and (**c**) resistance pulse sensing (RPS). The signals of (**B**) individual living *Pyramidomonas delicatula* cells: (**a**) chlorophyll fluorescence (CF); (**b**) side light scattering (SLS); and (**c**) resistance pulse sensing (RPS). The signals of (**C**) individual living *Isochrysis galbana* cells: (**a**) chlorophyll fluorescence (CF); (**b**) side light scattering (SLS); and (**c**) resistance pulse sensing (RPS).

In order to demonstrate that the developed detection device can detect the signals of CF, SLS, and RPS of a single microalgae cell simultaneously, the microalgae cells of *Dunaliella salina* were chosen as samples to be tested by the developed device. The signals of CF, SLS, and RPS of individual living *Dunaliella salina*, *Pyramidomonas delicatula*, and *Isochrysis galbana* cells are shown in Figure 4A–C, respectively. As can be seen from Figure 4A,B, and C(a), the CF pulse of a microalgae cell generates when the microalgae cell is passing through the detection spot. Every pulse of CF represents a living microalgae cell and the quantities of the CF pulses are equal to the quantities of the living microalgae cells. The amplitude of the CF pulse is positively proportional to the activity of the microalgae cell. For the microalgae cells of the same species with similar activities, the amplitudes of the CF pulses are closed and fluctuate around an average value. At the same time, the SLS pulse of the microalgae cell also occurs when the microalgae cell enters the detection area, which is shown in Figure 4A,B, and C(b). Every SLS pulse is corresponding to a CF pulse and the amplitude of the SLS pulse is related to the surface roughness and intracellular contents of the microalgae cell. Meanwhile, the RPS pulse also occurs when the microalgae cell is moving through the sensing gate, which is shown in Figure 4A,B, and C(c). The RPS signal lags behind the other two signals of CF and SLS with a slight delay. This is related to the position relationship between the photodetectors and the sensing gate in the microfluidic chip. If the sensing gate in the microfluidic chip is placed in front of the photodetectors, the RPS pulse will generate ahead of other two pulses, otherwise RPS pulse lags behind. Thus, it can be seen that the signals of CF, SLS, and RPS of a microalgae cell can be detected simultaneously by the developed device when the single microalgae cell is passing through the detection spot. 

**Figure 5:**
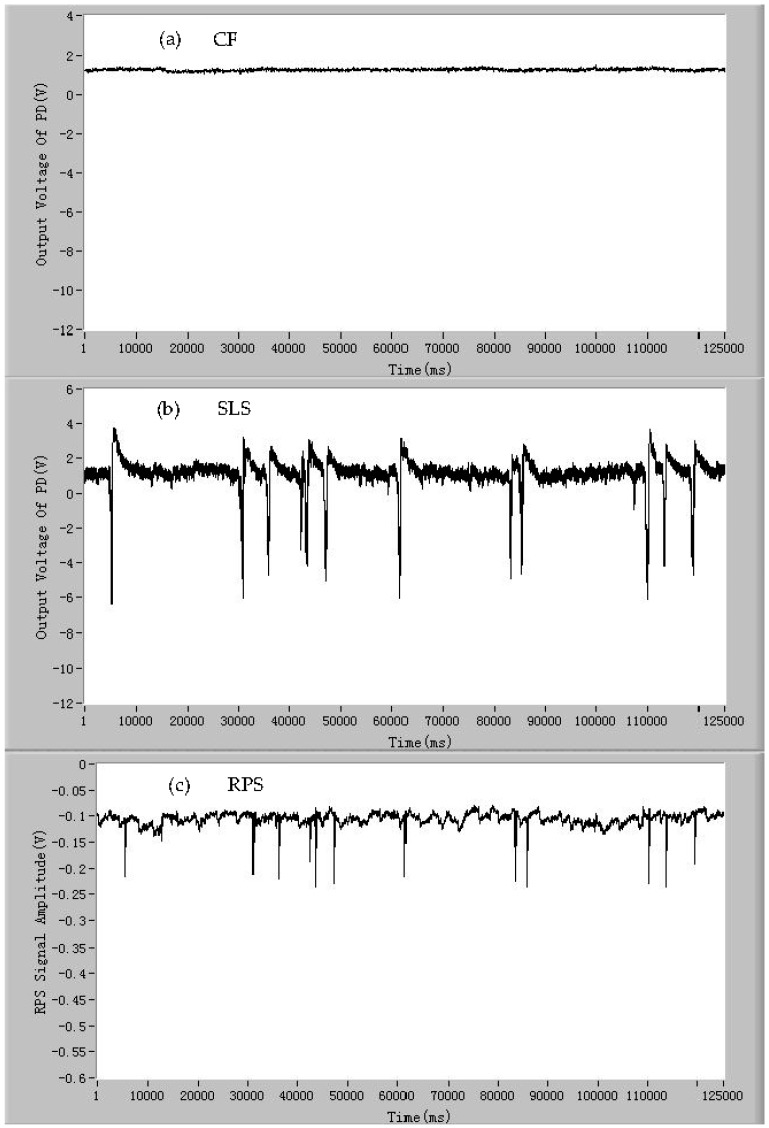
The signals of individual dead *Dunaliella salina* cells: (**a**) chlorophyll fluorescence (CF); (**b**) side light scattering (SLS); and (**c**) resistance pulse sensing (RPS).

**Figure 6:**
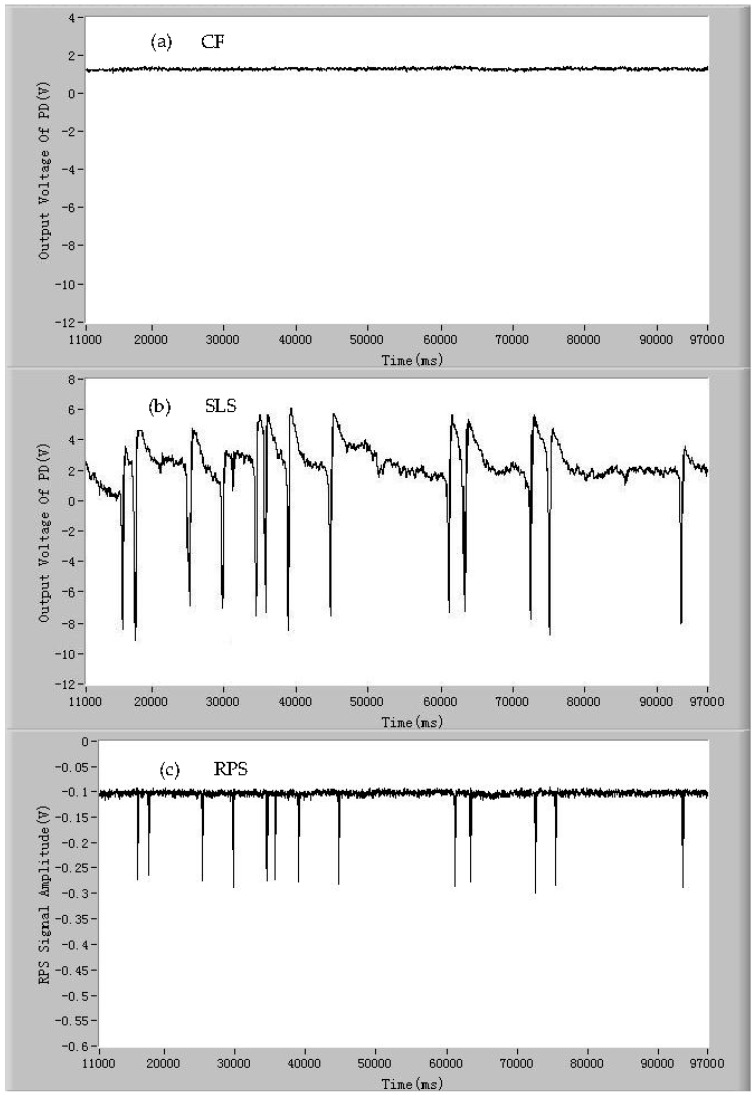
The signals of individual 10 μm polystyrene particles: (**a**) chlorophyll fluorescence (CF); (**b**) side light scattering (SLS); and (**c**) resistance pulse sensing (RPS).

In addition to the living microalgae cells, there are many other impurities in ballast water, which are not the ones that are concerned in ballast water detection. To investigate if the detection device can distinguish between the living microalgae cells and other microparticles, dead *Dunaliella salina* cells and the polystyrene particles were used as samples to be also tested by the developed device. The results are shown in Figure 5a–c and Figure 6a–c, respectively. The process of the inactivation treatment of the microalgae cells has been stated before. From Figure 5a, it can be seen that when a dead microalgae cell passes through the detection spot, no CF pulse generates. This is because the photosynthetic electron transport chain was destroyed under the heat treatment and the microalgae cells lost the capacity of photosynthesis and, thus, no chlorophyll fluorescence emits from the microalgae cells. The pulses of SLS and RPS generate simultaneously, which are shown in Figure 5b,c, respectively. Compared with the signals of SLS and RPS of the living microalgae cells, the average signal-to-noise ratio of these two signals of dead microalgae cells declined slightly. The declines are possibly related to intracellular degradation. The signals of CF, SLS, and RPS of the polystyrene microsphere particles (~10 µm in diameter, Sigma-Aldrich, St. Louis, MO, USA) are shown in Figure 6a–c. It is obvious that there are no CF signals from the polystyrene particles. The signals of SLS and RPS of the polystyrene particles were detected simultaneously. The average amplitude of RPS pulses of the polystyrene particles are larger than that of the *Dunaliella salina* cells owing to the difference of the average size. More significantly, the average amplitude of SLS pulses of the polystyrene particles are much larger than that of the microalgae cells because a larger difference in the surface roughness and contents in the microparticles exists between the polystyrene particles and the *Dunaliella salina* cells. The above experiments show that the developed device can distinguish between the living microalgae cells and other impurity particles, such as the dead microalgae cells and the inanimate particles, by the signals of CF, SLS, and RPS.

### 3.2. The Classification of Species by Signals of CF, SLS, and RPS of the Mixed Microparticles

**Figure 7:**
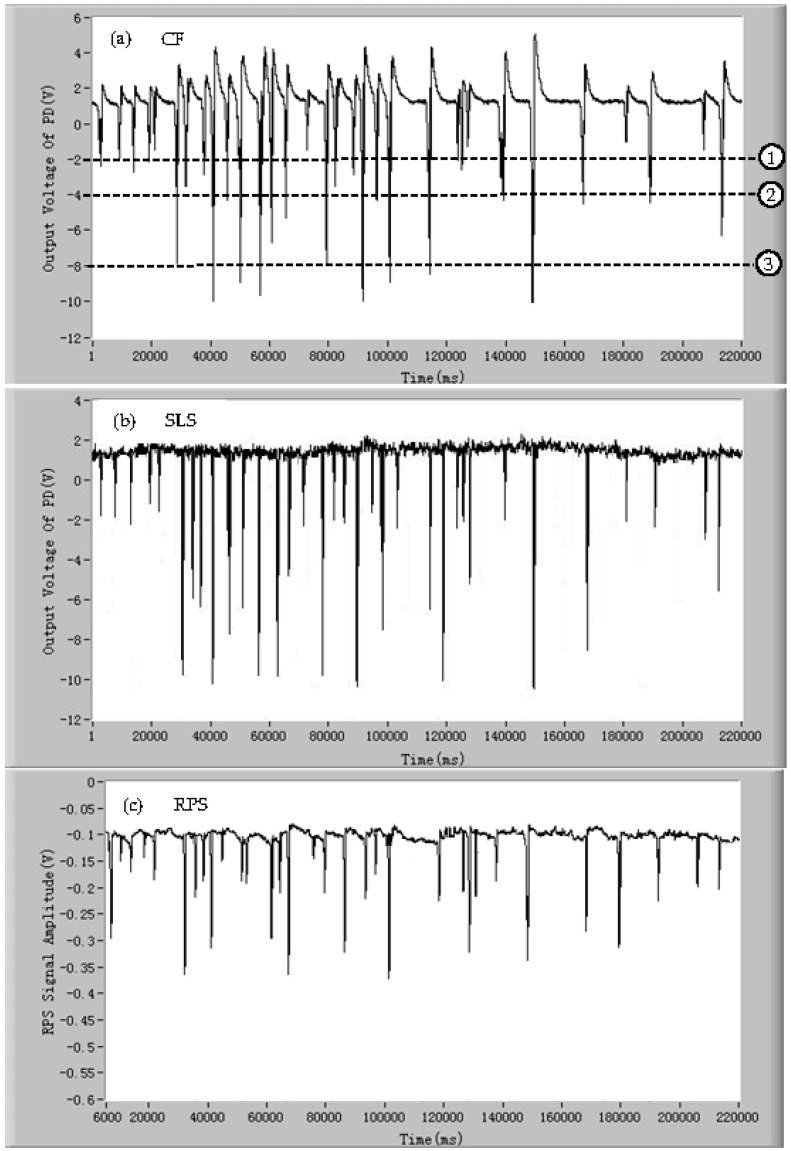
The signals of the CF of three mixed microalgae cells (*Isochrysis galbana*, *Dunaliella salina*, and *Tetraselmis chui*): (**a**) chlorophyll fluorescence (CF); (**b**) side light scattering (SLS); and (**c**) resistance pulse sensing (RPS).

To investigate the performance of detection of the mixed microparticles by the developed device, three kinds of microalgae cells (*Isochrysis galbana*, *Dunaliella salina*, and *Tetraselmis chui*) were mixed as samples. In order to analyze the principle briefly and not lose generality, the signals of CF of the microalgae cells were taken as examples, and the CF signals of the mixed microalgae cells are shown in Figure 7. From the results, it can be seen that the average amplitudes of the CF pulses of three microalgae species cells are different, which are easy to be separated and marked by the dash line with numbers ①, ②, and ③ in Figure 7. By the amplitudes of the CF pulses of the single microalgae cells, the microalgae cells can be divided into the different groups. The other two signals of SLS and RPS of the microalgae cells have also the similar characteristics; that is, the same or similar microalgae species cells have similar characteristics of SLS and RPS pulses.

### 3.3. Contrast Experiments between the Developed Device and the Commercial Flow Cytometer

**Figure 8:**
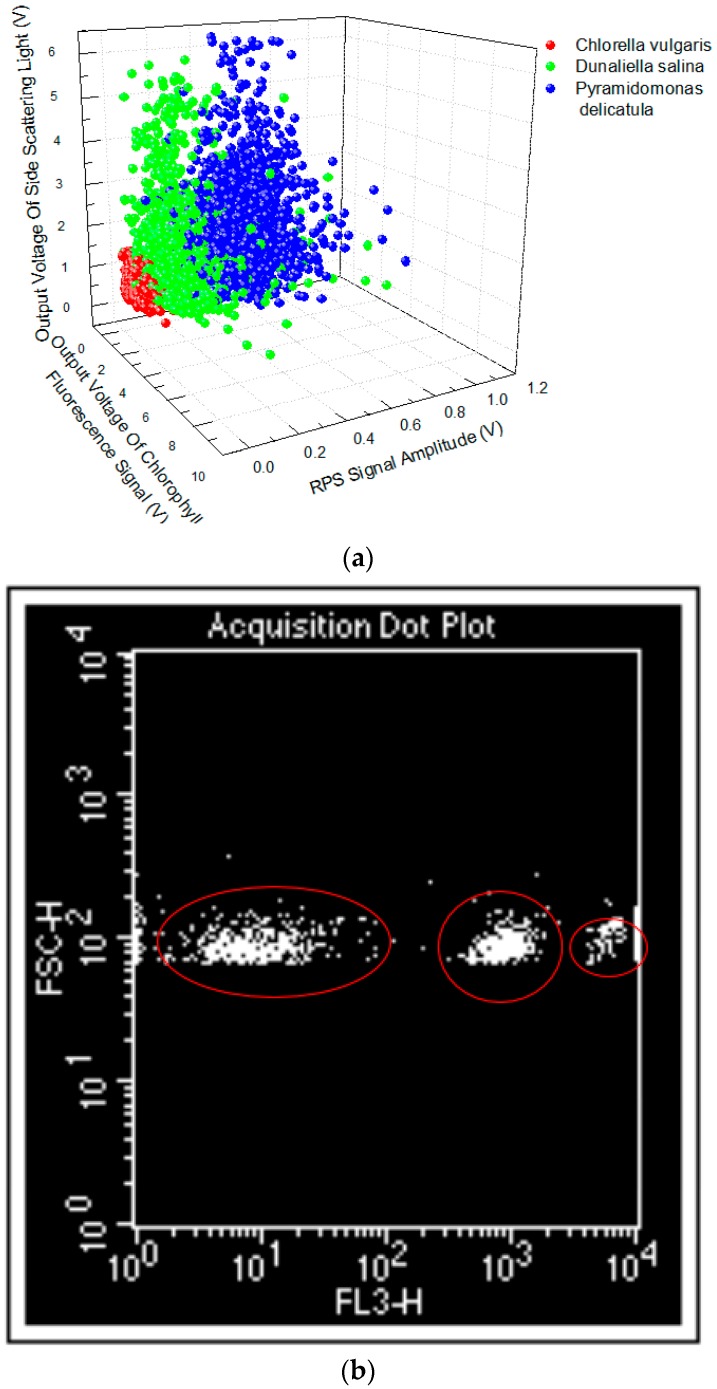
The comparison experiments of the classification of the microalgae cells between (**a**) the developed device and (**b**) the commercial flow cytometer.

Based on the above analysis, a microalgae cell can be classified by the amplitudes of CF, SLS, and RPS pulses of the microalgae cell. To demonstrate the performance of the classification of the microalgae cells of the developed device, three microalgae species (*Chlorella vulgaris*, *Dunaliella salina*, and *Pyramidomonas delicatula*) were used as samples and contrast experiments between the commercial flow cytometer and the developed device were conducted, with the results shown in Figure 8. Two- and three-dimensional distributions of the microalgae species cells are given from the flow cytometer and the developed device, respectively. Three kinds of microalgae cells are divided clearly into three groups by the commercial flow cytometer and the developed device. The results show that there is a good agreement between the commercial flow cytometer and the developed device. Based on the principle of simultaneous detection of the CF, SLS, and RPS signals of single microalgae cells in a microfluidic chip, the developed compact device can identify the classification of the microalgae cells accurately. More importantly, the developed device is compact, portable, and low cost. In addition, three-dimensional characteristics of the microalgae cells may provide a greater fine classification of the microalgae cells.

### 3.4. Limit Analysis of Classification

The minimum among these three signals of CF, SLS, and RPS of the microalgae cells may constrain the limit of detection (LOD) of this device. Though the different microalgae species have different chlorophyll, intracellular contents, and surface roughness and sizes, in general, the amplitudes of the signals of CF, SLS, and RPS all decrease with the decrease in the size of the microalgae cells. The size of the microalgae cells can become a parameter to evaluate the LOD of the developed device. For the present developed device, the microalgae cells with a diameter of about 2–3 µm can be detected. At this time, the CF signal becomes the minimum of three signals, while the LOD of RPS can reach about several hundred nanometers and LOD of SLS is about 1–2 µm. Some feasible methods can also be taken to further improve the limit of detection, such as a smaller sensing gate, greater power of excitation light, more sensitive photodetectors, and so on. 

## 4. Conclusions

A novel microfluidic device for the classification of microalgae cells was presented in this paper. The new features of the developed device include: (1) the physiological characteristics of microalgae cells corresponding to the signals of CF, SLS, and RPS were analyzed simultaneously to identify the classification of the microalgae cells, which include activity, surface roughness, intracellular contents, and size; (2) simultaneous detection of three signals of CF, SLS, and RPS of single microalgae cells on a disposable fiber-free microfluidic chip was accomplished; (3) the detection system was integrated in a compact device of about 18.5 cm × 8.5 cm × 12.3 cm and a weight of 720 g; and (4) the developed device has the advantages of accuracy, quickness, portability, and low cost, which are especially suitable for rapid on-site detection of ship ballast water.
